# The Challenges Facing VCFSEs in Integrated Care Systems: A Qualitative Case Study of a Unitary Authority in England

**DOI:** 10.5334/ijic.9072

**Published:** 2026-05-14

**Authors:** Catherine Leyshon, Michael Leyshon, Shukru Esmene, Miriam Leyshon, Olly Clabburn

**Affiliations:** 1 Centre for Geography and Environmental Science, University of Exeter, Penryn, Cornwall, UK; 2 Quay Research, London, UK; 3 Independent researcher, UK

**Keywords:** Voluntary and Community Sector, integrated care system, commissioning, substitution, system maturity

## Abstract

**Introduction:**

Integrated Care Systems (ICSs) in England seek ever-closer collaborative relationships between health and social care providers, local authorities, and the Voluntary, Community, Faith and Social Enterprise (VCFSE) sector to deliver public services in place. However, ICSs present significant challenges for VCFSEs.

**Methods:**

In-depth qualitative case study of VCFSEs in an ICS contiguous with a Unitary Authority in England.

**Results and Discussion:**

i) the role of the ‘microbiome’ of smallest VCFSE in the ICS is unclear; ii) current commissioning models work against the effective participation of VCFSEs in ICSs; iii) short-term and under-funded contracts threaten VCFSEs; iv) substitution and appropriation represent tangible threats to VCFSEs through increased demand and bureaucratisation; v) the degree to which ICS support a vibrant, heterogeneous, and sustainable VCFSE sector depends on ‘system maturity’.

**Conclusions:**

Integration is a process, not an event. The evolving landscape of service design presents both opportunities and challenges for VCFSEs, requiring careful management to harness strategic advantages while addressing operational risks.

## Introduction

The landscape of public service design and delivery in Western economies is evolving in response to the ever-increasing demands for stretched services. Cuts to funding and shocks to global systems, such as Covid have exacerbated structural inequality, leading to areas of deprivation that exhibit chronic health inequalities, especially poor mental and physical health [1]. At the same time, and partly in response, there has been a drive globally towards the devolution of responsibility, decision-making, and budgets, and attempts to improve the transparency, efficiency, and accountability of public services [2]. Meanwhile, new paradigms of place-based delivery are being implemented across the globe [3] with one example being Integrated Care Systems in England [4], which seek ever-closer collaboration between diverse providers from the health and social care sector, local authorities, the private sector, and voluntary, community, faith, and social enterprises (VCFSEs)^1^ to deliver public services in place.

This paper answers this research question: *what are the challenges facing VCFSEs in the new landscape of service design and delivery associated with the formation of integrated care systems?* This question is relevant in the UK, where some of the challenges have persisted for decades and deserve fresh scrutiny, and globally. As health systems in the USA, Canada, New Zealand, Australia and Europe [5,6,7] develop models for integrated care, there is a drive to involve VCFSEs. Our empirical focus is on England, but many countries grapple with achieving meaningful collaboration, which requires attitudinal shifts, new working methods, rebalancing of power relations, sufficient resources, creative solutions for long-standing barriers and misunderstandings, and improved shared learning [8,9,10]. We explore these issues through an in-depth qualitative enquiry into the role of VCFSEs in an Integrated Care System contiguous with a Unitary Authority in England (a tier of local government covering whole or part of a county or large town or city). Our findings are not only relevant to the UK but to anywhere the participation of VCFSEs in integrated care takes place.

The case study research underpinning this paper was co-designed and completed with a VCFSE local infrastructure organisation (LIO). There are 519 LIOs in the UK, which represent the sector locally and support networking, training, leadership, and knowledge exchange [4,11,12]. We review the literature on the voluntary sector and ICSs before discussing our methods. The results are presented thematically, covering the composition of the VCFSE sector, commissioning, funding, substitution and appropriation, and system maturity.

## Integrated Care and the Voluntary Sector in England

Integrated Care Systems (ICSs) are partnerships that bring together health organisations, local authorities, and others to take collective responsibility for planning services, improving health, and reducing inequalities across geographical areas [13,14]. There are currently 42 ICSs across England, each covering 500,000–3 million people. Some ICSs have existed informally since 2016, but the 2022 Health and Care Act gave ICSs statutory powers and responsibilities [6]. ICSs in England are run by Integrated Care Boards (ICBs): local (executive) partnerships with complicated committee structures comprising the different stakeholders. ICBs plan and deliver health services to local populations [4], though this has not been straightforward. The imminent abolition of NHS England, 50% cuts in funding, clustering of ICSs into much larger units, and local government reform will introduce further perturbation.

ICSs are part of a trend towards a holistic model of service design and delivery that envisions local service providers, VCFSEs, local government, and private-sector agencies working together to deliver health services and programs [15]. A recent study noted that ICSs should go beyond this remit by building on community assets that have been overlooked or undervalued by governments, such as cultural capacities, social networks, and natural resources – including those run by VCFSEs [16]. Utilising these assets promises greener, more sustainable care services, a deep-rooted ethic of care in communities, and resilience in the face of societal challenges [17,18,19,20]. VCFSEs deliver effective support through locally coordinated services [17], enhancing service efficiency and creating a ‘more beneficial reality for patients’ [21: 2]. VCFSEs ‘secure ordinary living [by] organising education and entertainments, arranging transport, enhancing existing services, suggesting alternative solutions to healthcare and advancing public health through health promotions’ [22: 258].

Yet, there is still uncertainty about how to ‘implement policy advocating effective involvement of [VCFSEs], particularly in healthcare systems where complex and diverse goals, priorities and values are negotiated’ [23: 549]. The challenges associated with integrating VCFSE practices and expertise into care systems are not unique to the UK. VCFSEs face ‘radically heightened expectations’ resulting in ‘qualitative changes in their role’ [2: 2]. Some VCFSEs may benefit in terms of strategic direction, continuity of service provision, and financial stability, but there are also significant risks. Not enough is known about the meaning and practice of integration or the way it is shaped by current assumptions, values, and priorities on all sides, especially where lived experience and diverse expertise are sidelined. Further, the degree to which integration is attentive to the heterogeneity of the voluntary sector is not clear: different VCFSEs require different levels of, and approaches to, integration. Small-scale voluntary organisations delivering community activities (e.g. chat cafes, hobby groups, community fridges, support for mental health, *inter alia*) are different to LIOs or voluntary sector alliances with some permanent staff participating in service delivery, strategy discussions, and commissioning. They all tackle health inequalities [24] and the social determinants of health [25], but must be understood as ‘set apart from both the informal, intimate social relations of family and friendship but also from the state itself’ [26: 920]. Being organic and dynamic enables VCFSEs to be highly adaptable, flexible and responsive [27]. But these qualities are also a source of tension, particularly when the rapid integration of VCFSEs into local health and care systems is considered. Before unpacking this further, we now outline our methods.

## Methods

We adopted an instrumental case study approach, appropriate for generating an ‘in-depth, multi-faceted understanding of a complex issue in its real-life context’ [28: 1], namely the challenges facing VCFSEs operating in ICSs [23]. Our case study is an ICS in a rural unitary authority in England, serving a population of c.500,000 people.

We undertook a desk-based review of policy documents, reports from think tanks, national VCFSE organisations, and academic papers. Thirty-two one-to-one and three group semi-structured interviews were conducted with representatives of VCFSE organisations from the smallest (two people) to the largest (turnover in the millions) (Table 1) and with members of three voluntary sector alliances (these are strategic, thematic groups, which share challenges and opportunities, provide peer support, lobby, and collaborate with other ICS partners). Semi-structured interviews help participants express their perspectives and generate rich data while keeping the insights aligned with a study’s aims [29]. A non-probability (purposive) sampling strategy [30] was used, and VCFSEs providing a wide range of services and activities, from arts-based groups to home visits by carers, participated (Table 2). Informed consent was obtained from all interview participants.

**Table 1:** Participants by size of organisation.


SIZE (BY INCOME)	PARTICIPANTS

Micro	1

Small	10

Medium	7

Large	7

Major	1

Information not available	2

**Total**	**28**


**Table 2:** Participants by type of organisation.


SERVICES	PARTICIPANTS

Community Activities	7

Children and Youth	6

Care	3

Environmental	3

Mental Health	3

Children and Family	2

Faith	2

Disability	1

Infrastructure Organisation	1

**Total**	**28**


The paper also uses anonymised free-text comments from the 2024 State of the Sector Survey, which was conducted by the Local Infrastructure Organisation. These added depth and breadth to the interview data. The free-text comments and interview transcripts were analysed using discourse analysis, which asks not only how many times a topic was raised but how it was spoken about. Discourse analysis has been underutilised within healthcare system research [31], although its use is increasing in research on health and care settings [32]. An inductive coding framework was produced by repeatedly assessing the data for patterns and assigning codes accordingly [33]. The following themes were identified:

- Commissioning
- Funding and sustainable investment
- Substitution and appropriation

Two cross-cutting themes also emerged: how the ‘microbiome’ of smallest organisations can be nurtured and ‘system maturity’.

Ethical approval was granted by the University of Exeter Faculty of Earth and Environmental Science Ethics Committee (#6679034).

## Results and Discussion

### Nurturing the microbiome

A common way to differentiate between VCFSEs in the UK is by size measured by annual income [34]. Table 3, based on the most recent data from the National Council of Voluntary Organisations (NCVO), shows that 80.34% of the sector are micro or small organisations with an income between £0 and £100,000 [34]. These organisations form the all-important ‘microbiome’, which is largely invisible but essential to a healthy system [35] in the same way as our gut flora and countless other micro-organisms contribute to the functioning of the whole human bio-physical system. The metaphor of the ‘microbiome’ recognises both the number and importance of the smallest voluntary sector organisations that are critical to the functioning of society, filling gaps that few know exist and operating with small amounts of money. The concept is applicable in any national setting [10,36].

**Table 3:** Number and percentage of voluntary sector organisations by size, 2020/21 (NCVO, 2023).


INCOME	CATEGORY	NUMBER OF ORGANISATIONS	% OF ALL ORGANISATIONS

< £10,000	Micro	77,295	47.14

£10,000-£100,000	Small	54,431	33.20

£100,000-£1m	Medium	25,569	15.59

£1m-£10m	Large	5,861	3.57

£10m-£100m	Major	743	0.45

> £100m	Super-major	61	0.04

**All organisations**	**Total**	**163,959**	**100.00**


Despite being very under-researched in the UK and internationally, thinking about the smallest VCFSEs as a microbiome helps in two ways. First, policymakers, health and care commissioners, and ICBs deploy systems thinking: an approach that avoids isolating a problem to one factor and understands it as the outcome of many factors. Fully embracing systems thinking means being as attentive to the smallest parts of the system – the microbiome (hard to see but essential) – as the largest (e.g. the NHS and the local authority). Second, the metaphor of the microbiome calls attention to the ‘rich and thriving sector of informal grassroots organisations operating below the usual regulatory and administrative radars’ [37: 4]. In England, some VCFSEs are visible via Charity Commission and other data, but in general, the quality of data on the smallest voluntary sector organisations is poor [38].

A 2024 survey in our case study area (citation omitted for anonymity) suggests 47.1% of respondents consider themselves in the micro or small income categories (<£10k and <£50k a year). Ten percent fall into the second ‘small’ category (<£100k). Only 24.62% of respondents – mainly large organisations – hold contracts to deliver statutory services in the ICS. Smaller VCFSEs employ various strategies to operate outside of the formal commissioning and contracting mechanisms, such as:

- Diversifying income through fundraising events, crowdfunding, and selling products/services – but these efforts sometimes reduce the capacity for service delivery.
- Seeking grants and alternative sources of funding. Some people spend their own leisure time preparing grant applications.
- Collaborating or sharing resources with other organisations for joint bids.
- Utilising volunteers for administrative tasks related to funding.
- Exploring partnerships with local businesses, councils, and infrastructure bodies for support.

However, many feel that more systemic solutions are needed, such as:

- Longer-term, flexible funding models covering core costs.
- Centralised support for legal services, HR, and funding advice tailored to VCFSEs. This would usually be provided by the LIO, highlighting the importance of adequately funding LIOs.
- Streamlined application and reporting processes.
- Greater recognition of the value and impact voluntary organisations provide, without necessarily using the measures currently preferred by funders or commissioners in the ICS.

This last point raises issues with commissioning, to which we now turn.

### Commissioning

Despite a decade of experiments in integrated care

…it is still unclear how local healthcare commissioners allocate budgets to healthcare services, particularly in the context of integrated care [or] what factors drive the local decision-making process, who is involved, and what are the main challenges that commissioners face when investing in one intervention over others [39: 2].

Although focused on healthcare commissioning, these comments ring true of commissioning in ICSs more generally, particularly when VCFSE organisations are involved. VCFSEs are widely viewed as an ‘innovative, nimble and flexible sector which is embedded in, and reaches out to, communities and particularly hard-to-reach groups’ [2: 6], cost-effectively providing innovative programmes and services with positive impact on community social capital [2]. However, third-sector participation in public services does not occur on a level playing field [40]. Commissioning processes can fail to ‘recognise the added value that small and local organisations can bring’ [41: 105–6], including local knowledge and support, additional resources, flexibility, and commitment. Some VCFSEs do not always have the capacity, capability, infrastructure, expertise, or willingness to deliver public services [2]. The UK’s Civil Society Strategy argues that the current public service delivery model favours large companies who can navigate complex commissioning systems, carry risk, and bid competitively [41], sometimes muscling out smaller, local organisations [42].

Participating in commissioning requires VCFSEs to have an understanding of the health and care system itself, and how the needs for statutory services are identified and funded. One interviewee pointed out:

‘… the system has capital letters. So it’s ‘The System’ and you’re either in it or you’re not. And we’re very much embedded into that as a voluntary sector partner.’ – Large VCFSE offering commissioned services.

The consequences of not being embedded are clear to many of the smaller organisations that we interviewed: larger organisations have more capacity for writing applications for commissioned work and they have a close working relationship with commissioners. This leaves the area’s micro and small organisations feeling disillusioned about integrated care.

In our case study area, this issue is illustrated in the arrangements for one important area of delivery (not identified for reasons of anonymity). There are two strong narratives: i) that one provider dominates the delivery, to the perceived detriment of the service overall; and ii) attempts at a more collaborative approach through two big initiatives have not succeeded in either providing better support for the client group or distributing funding more equitably amongst the diverse group of VCFSEs operating in this space. Smaller voluntary sector organisations express frustration that one large provider receives a disproportionate amount of funding and referrals from statutory services, despite offering a limited range of support to the client group. Smaller providers outlined that this situation works against a holistic, person-centred model of delivery. Several interviewees reported that the largest provider, finding they cannot meet a client’s needs, refers to smaller organisations without any transfer of funding. One interviewee expressed the views of others when they reflected on how, despite not having funding, they will not turn people away because of the impact on their clients of doing so:

‘If you’re scrambling around at the lower decks of the voluntary sector … and just little contracts and small amounts of money and just keeping your head above water, and you get another [client] come with no money, but you want to be able to respond, but you’ve got to go ‘can I actually respond?’ …it’s soul-destroying. Massively traumatising.’ – Small VCFSE offering community activities.

Calls for commissioning to be more collaborative, involving input from various providers on local needs, are militated against by the existence of back-channels through which extramural conversations *‘off the radar’* take place, building relationships between providers and commissioners. The same interviewee went on to say:

‘There is a wider appetite, I think, for trusted organisations to occupy more space.’

As a result, when commissioners have resource:

‘It is easier and psychologically safer for them to come to myself or [other local providers] … It becomes a kind of shorter route, which …is really positive, because we have got trusted deep relationships in our sector … what that potentially does is shut the door on other people that might be interested in accessing some of that resource or taking part of that contract.’

Commissioning arrangements are bespoke to specific countries’ public sector funding models. Nevertheless, actions that promise to energise, mobilise, and create capacity in the VCFSE sector are generalisable. Getting the commissioning process wrong presents operational and existential risks to VCFSEs [42] (Table 4). Despite being anticipated by policy research spanning back to 2018 [43], our interviewees also raised these issues.

**Table 4:** Challenges faced by VCFSEs involved in commissioning and public service delivery [42 and interviews].


OPERATIONAL CHALLENGES	EXISTENTIAL CHALLENGES

Accessing and maintaining position within the commissioning system. Tendering, track record, quality assurance systems, balance sheets. Doubts over managerial and operational capabilities. Demanding output or outcome expectations. Full cost recovery.	Commissioners determine VCFSE activities. Threats to the distinctive qualities of the sector: Independence, collaborative ethos, flexibility. Advocacy, commitment to mission. Formalises relationships with service users. Risks mission drift. Intensifies competition.


A key challenge is to ensure that integration of the VCFSE sector with public services and commissioning processes acknowledges and respects the sector’s heterogeneity. This is reflected in the Department for Culture, Media and Sport’s action plan to engage VCSFEs in its supply chain [44], which identifies supply-side and demand-side barriers to participation in procurement, including the capacity to deliver contracts and lack of understanding by commissioners of the VCFSE sector.

Conditioning these debates about VCFSEs and commissioning is the rhetoric and practice of integration. The King’s Fund notes that ICBs have been born into difficult times [45]. The work of establishing new systems and cultures that promote prevention, collaboration, inclusion, and community-based models of care is ongoing. Attempts to open a space of innovation in service design and delivery can become secondary to addressing performance challenges within the local authority and acute care sector. The King’s Fund’s 2020 report, *Thinking Differently about Commissioning*, outlines the challenges of transforming commissioning after nearly 30 years of quasi-market arrangements. These challenges include:

- Developing relationships and changing ingrained behaviours, including pooling sovereignty, giving up power, and committing to transparency and consensus decision-making processes.
- Identifying effective leadership, role-modelling, and senior-level commitment across the system.
- Adjusting procurement approaches and local incentives to support rather than inhibit collaboration.
- VCFSEs, the independent sector, patients, and the public become key partners in collaborative approaches to commissioning, utilising the unique strengths of smaller, local organisations [46].

Transforming commissioning might also entail exploring alternative models, wherein the exact service specification could emerge through community-level interaction and experience [47], wider redistribution of power and resources from the public sector to communities [48] and/or creating Single Point of Commissioning (SPOC), where a single organisation is responsible for commissioning services across multiple sectors.

We now turn from questions about commissioning structures to examine funding and sustainable investment in the sector.

### Funding and sustainable investment

Across Western economies, funding of the third sector is contentious, reflecting concerns about resource distribution, fairness, value for money, quality assurance, governance, and reporting [36]. The VCFSE sector in England is funded via a mixed portfolio of grants, donations, fundraising events, trading, membership fees, endowments, investments, and contracts or commissioning via local authorities and the NHS [49]. Despite playing a critical role during the Covid-19 pandemic [18], VCFSEs have since struggled with funding [50]. Since 2007/8, VCFSE income has fallen 35% from £3.1bn to £2bn [34]. The developing partnership between local government and the voluntary sector and the increase in the proportion of VCFSE funding that comes from local government [51], make finances vulnerable in several ways, explored below.

#### Less money in the system

Many interviewees and State of the Sector survey respondents noted that the system has less funding to meet demand. As one large organisation surmised, *‘The pot of gold is so small’*. Local Authorities are a significant player in ICSs and an important funder of VCFSEs, providing 13p in every £1 through grants and contracts [52]. However, there has been a ‘revolutionary change’ in local authority financing in England since 2009/2010 [53: 2062]: spending has fallen and changes to the funding system mean that councils are more dependent on local tax revenue. Whilst spending on statutory services has suffered the smallest cuts, discretionary spend has been reduced significantly [52]. At the same time, the cost-of-living crisis has increased demand on public service providers and VCFSEs, presenting four key threats to the voluntary sector through increased running costs, salaries, interest rate rises and lower public donations [49].

In the 2024 survey of VCFSEs in our case study area (citation omitted for case anonymity), 73.11% of respondents agreed strongly or agreed with the statement that funding was harder to find. Meanwhile, the top 6 impacts on the area’s VCFSEs from the cost-of-living crisis are increased demand, cost of lighting, heating, equipment, and supplies, and decreased funding from public sources and private donations.

Less money in the system means greater competition in two linked ways. First, a quasi-market has emerged which emphasises competition between organisations, damaging trust and collaboration between VCFSEs [54]. Second, more organisations are chasing smaller grants. In our case study’s VCFSE survey, 80.51% of respondents agreed strongly or agreed with the statement that there is more competition for funding. The challenges of demonstrating impact and value to funders, especially for smaller organisations, increase the pressure.

Our interviewees reported spending time searching for and applying for small amounts of money multiple times every year, increasing competition:

‘…everybody [is] scurrying around to try and grab limited resource … internal [county-based] money or external grant base or national money, and it becomes a bit of a bonfire, and actually nobody wins in that case, because it just becomes fragmented … and everybody gets terribly precious about trying to do their thing’ – Small VCFSE offering community-based services.

Financial existence is a struggle for smaller organisations – particularly those which are volunteer-led or have a small workforce with limited capacity [55]. Larger organisations are better suited to dealing with the tasks and processes associated with delivering public services in the ICS [40]. In our case study area, one interviewee noted:

‘The large organisations… they’ve all got people whose job is to … plough through that stuff and make it happen. And they’ve got the time, they can invest in that, and they’ve got the reserves that they can invest in that as well … we can’t compete with that.’ Small VCFSE offering community-based activities.

This creates a looming existential crisis for the sector’s micro and small organisations, impacting the degree to which micro and small organisations can deliver the place-based support valued and sought by ICSs. This issue is compounded by underfunded and short-term contracts.

#### Underfunded and short-term contracts

A UK-wide State of the Sector Report for 2024 identifies the risk that underfunded contracts held by charities risk undermining the delivery of essential public services [51]. It is common for VCFSEs to be viewed as more cost-efficient than the statutory sector and able to access sources of funding not available to statutory agencies [2]. However, VCFSEs should not be viewed as a cheap or, in some cases, free resource [2]. One VCFSE reflected the views of many:

‘We were commissioned to run some [XXX] schemes [by the local authority]. We were asked to put a bid in … we [were] then told it was too high. So we had to reduce it, probably by about a quarter … And then this is a piece of work that we’re still doing. And what we’re finding is we’re having to go and seek funding to be able to continue that. So we still get offered some financial contribution from [the local authority] for that, but we’re having to go to funding and get the rest of the money to be able to deliver that to what we would deem as satisfactory quality’ – Small VCFSE offering community-based services.

Participants also referred to how funding streams rarely cover wider costs that VCFSEs face when providing services:

‘Obviously, it’s a voluntary sector, so you can’t pay them [volunteers] for their time, but it’s not free … For instance… transport isn’t cheap. So to be able to… to give [a volunteer] something for [their] travel will make it more likely [they’ll] be able to come. Because not everyone can afford to spend five pounds a week on transport to something that they don’t necessarily need to do.’ – Small VCFSE offering community-based services.

Such issues are compounded by short-term funding, which magnifies the sector’s fragility and precarity [56]: services are created only to disappear a couple of years later. Further, fixed-term funding engenders a focus on fulfilling the terms of the grant. These conjoined risks are expressed by this group interview participant:

‘I think we’re measuring outcomes… which is right, but it needs to be connected to people in order for us to see that whole value in the community and that wider perspective and the longevity of it rather than just ‘well we’ve had the year [long] project. Wasn’t that marvellous?’ No, it wasn’t, because we’ve all gone back to nowhere… Where it’s five-, six-year projects, it’s actual investment in the community, not project’ – Group Interview Respondent.

Less money in the system means that the sector remains in response mode and is unable to innovate in service design and delivery within the ICS, as one interviewee noted:

‘If I thought we were going to attend a meeting where we could innovate, be strategic… Then I’d happily put time into this. But we’re not seeing any movement at all from the Council or the ICB. At the moment, I’m not convinced it’s at all worthwhile for us to be working with them’ – Large VCFSE offering faith services.

### Substitution and appropriation

Defined by a commitment to mutuality and service, operating independently and driven by a compelling social and civic mission, VCFSEs in many developed economies have a long history of ‘filling the gaps’ left by the market or the state [57]. This section looks at *substitution* and *appropriation*, which describe different dynamics in the relationship between VCFSEs and the ICS.

Substitution is what happens when VCFSEs deliver services traditionally provided by government, becoming a ‘safety net’. Since the 1980s, the scale and scope of VCFSEs as a vehicle for service delivery have increased due to cuts in public spending, policy shifts, shrinking welfare state, and changes to public health and social care provision [57,58]. In this ‘shadow state’, delivery, risk, and responsibility have been devolved to non-state actors to fill the gaps in welfare provision [59]. Impacts on VCFSEs include increased demand, sometimes without a corresponding increase in funding, resulting in financial and operational stress.

For the service user, the quality and accessibility of services may vary, especially where providers are working in a specific area as large geographically as our case study area. Substitution raises concerns about accountability and the maintenance of standards, as voluntary organisations may not be subject to the same oversight as public sector bodies [60]. Whilst substitution has a long history, an emergent trend is towards appropriation. In ICSs in the UK and elsewhere, a closer working relationship between government has seen increased bureaucratisation, greater expectations about service delivery, and control over client groups [2,26,61]. Appropriation refers to the integration or co-opting of voluntary sector resources, methods, or roles by the public sector, with the result that the sector is required to meet public sector standards of quality, consistency, accountability, governance, equity, and access. There are damaging consequences for ICSs, as one quote in the recent State of the Sector report affirms: ‘*They [ICS partners] don’t understand the additional work we have to do and want us to work like they do, this is challenging, unproductive, and relationship destroying*’ (citation redacted for anonymity).

National government, local authorities, and the NHS wish to harness the VCFSE’s flexibility, responsiveness, and innovation through joint commissioning mechanisms, service design, delivery, and evaluation [62]. But whilst the rhetoric is about working in partnership, the power relationships are far from equal, and partnership can rapidly tip over into appropriation. A group interview respondent observed:

‘Sometimes when I’m talking to colleagues who are coming from a statutory perspective, they use the word collaborate, well, actually, they actually mean as a partnership, or a contract, where they’re the commissioner, and you’re doing what they want you to do.’ – Group Interview Respondent.

Some scholars argue that the third sector is being turned into a ‘governable terrain’ through narratives, strategies, and administrative and policy changes [62]. These impose a certain order on a diversely regulated group of organisations. This adds new administrative burdens and risks damaging the VCFSE’s impact on the people and communities it seeks to serve because, unlike the market and the public sector, the work of voluntary organisations precludes comparative performance measures [60]. There are specific reasons why this is the case:

- Difficulties identifying suitable measures of success due to the long–term mission of voluntary organisations and the aspirational nature of their goals [63].
- The inadequacy of financial measures in capturing the social impact of VCFSEs [64].
- The diversity of virtues ascribed to VCFSEs – e.g. democratic value, trust, and the ability to generate social capital – challenge performance management regimes intended to assess VCFSE’s work [65].

As in many ICSs, VCFSEs (in our case study area) deliver both statutory and non-statutory services. One study notes that this ‘necessarily incorporates [VCFSEs] into the pervasive managerial, audit and performance management systems that operate in the statutory sector.’ [2: 2]. A positive outcome of this might be that the sector’s historical strengths of mutuality and service help make public services more responsive, democratically accountable, and relevant to all sections of society. But the downside is greater commercialisation, with the principles of service subsumed by contract and audit culture [2]. These processes can have significant implications for the independence, identity, and operational capacity of voluntary organisations.

### System maturity

The creation of ICSs represents an opportunity to transform service design and delivery in place, but whether this process will result in a sustainable VCFSE sector characterised by vibrancy and heterogeneity depends much on ‘system maturity’. The need for a ‘system-wide approach’ to delivery challenges in the public sector is frequently invoked [66]. However, taking a systems approach (embodied most obviously in ICSs) requires unpicking decades of policies, practices, processes, deeply ingrained thinking, and cultures of working that have contributed to significant levels of mistrust of the system by VCFSEs (as evidenced in the EU VIGOUR programme) [3]. Thus, it is important to reflect on whether the system itself is mature enough to fully embrace collaborative working at every scale – particularly when this involves collaboration between the local authority and a diverse VCFSE sector, including the LIOs, larger VCFSE organisations, and the ‘microbiome’ of small groups.

There are concerns within the VCFSE sector in our study area about whether the wider system has indeed reached sufficient maturity [66] and whether system leaders are ready to let go of traditional ways of working and hierarchical control to allow VCFSEs to flourish in this new model.

Specific issues raised across group and individual interviews include:

- Lack of trust and flexibility from statutory bodies like councils, with a tendency to micromanage and impose bureaucratic processes on VCFSEs.
- Lack of VCFSEs’ voice in key decision-making forums within the ICS, or token involvement that does not respect the knowledge and skills of the sector.
- Rigid commissioning approaches that don’t align with VCFSE’s ability to innovate and adapt to community needs.
- Entrenched power dynamics and lack of understanding about VCFSE’s value, knowledge, and expertise.
- Uncertainty about whether the system has the maturity and readiness for the cultural shift required for integration and co-production.

Whilst some progress has been made, much more work is needed in areas like building trust, relationships, and a shared vision for the ICS model to reach its full transformative potential, not only in our case study area but elsewhere [67].

What does system maturity mean in the context of the relationship between the ICS and VCFSE? Of importance is structural integration or the extent to which different parts of the system are linked and coordinated. The SCIROCCO has been used in several European countries to evaluate system maturity [68] and could be augmented with: i) a clear understanding of what all the parts of the system are (including a diverse voluntary sector), and what each can contribute to the design and delivery of public services; ii) a shared vision of what collaboration means and what it can achieve, paying attention to different priorities and expertise and supported by effective co-designed mechanisms that foster collaboration; iii) a system that is responsive to need, recognising that need might be expressed in ways that do not align with current assessment, measurement, or evaluation practices; iv) the space, resources and appetite for innovation. This can quickly become secondary to addressing performance challenges within the local authority and the acute care sector within the ICS.

## Conclusion

Integration is a process, not an event. The local VCFSE sector in any given country is essential to the delivery of successful integrated care because they fulfil a variety of roles, from augmenting public services to substituting and even contracting for statutory services, highlighting their flexibility and adaptability. Nevertheless, the evolving landscape of service design presents both opportunities and challenges for VCFSEs, requiring careful management to harness strategic advantages while addressing operational risks.

The issues outlined in this paper are likely to become more complex and acute with the announcement of significant structural changes to ICSs, local authorities, and NHS England in 2025. ICSs are being organised into geographical clusters of roughly 1.5m people, creating efficiencies in commissioning. Further, local government reform is introducing new devolved power arrangements in England and, in some cases, the creation of larger Combined Authorities. Meanwhile, NHS England – the executive non-departmental public body that runs the NHS – has been abolished on the grounds of overreach and inefficiency. Remaining budgets are to be cut by half and staff merged into the Department of Health and Social Care. These developments add yet more complexity to unravelling decades of policies, practices, processes, deeply ingrained thinking, and cultures of working that have contributed to significant levels of mistrust of the healthcare system by VCFSEs (as evidenced in the EU VIGOUR programme) [3]. The delivery of the NHS 10-year plan contains ‘plenty of the what but less of the how’ [69: 1], raising issues of how to deliver a national plan within changing ICS footprints. Such seismic changes not only consume a lot of capacity but also fundamentally shift working relationships, processes, and power relations in a way that is not currently clear.

In the face of these issues, it is necessary to adopt a reflexive approach to service design and delivery that uses the following questions as a touchstone: ‘what is the *ask* of VCFSEs?’ and ‘what is the *offer* to VCFSEs?’. Understanding the ask and the offer involves, first, ensuring that the VCFSE voice is not only strongly represented but also listened to and acted upon and, second, that the heterogeneity of VCFSEs is embraced as one of the sector’s key strengths. This will require more research about and for the voluntary sector, noted by Miller et al. 2025 [70]. Effective commissioning, progressive funding models, and a recognition of the importance of the ‘microbiome’ will also help realise the power and potential of VCFSEs in integrated care systems.

## Footnotes

In different countries, VCFSEs are variously referred to as the voluntary sector, the Third Sector, not-for-profit, voluntary welfare organisations *inter alia*.
